# Validation of a Cleanroom Compliant Sonication-Based Decellularization Technique: A New Concept in Nerve Allograft Production

**DOI:** 10.3390/ijms23031530

**Published:** 2022-01-28

**Authors:** Federico Bolognesi, Nicola Fazio, Filippo Boriani, Viscardo Paolo Fabbri, Davide Gravina, Francesca Alice Pedrini, Nicoletta Zini, Michelina Greco, Michela Paolucci, Maria Carla Re, Sofia Asioli, Maria Pia Foschini, Antonietta D’Errico, Nicola Baldini, Claudio Marchetti

**Affiliations:** 1 Oral and Maxillofacial Surgery Unit, IRCCS Azienda Ospedaliero-Universitaria di Bologna, 40138 Bologna, Italy; claudio.marchetti@unibo.it; 2 Department of Biomedical and Neuromotor Sciences, University of Bologna, 40136 Bologna, Italy; viscardopaolo.fabbr2@unibo.it (V.P.F.); sofia.asioli3@unibo.it (S.A.); mariapia.foschini@unibo.it (M.P.F.); nicola.baldini@ior.it (N.B.); 3 BST Biomedical Science and Technologies Lab, IRCCS Istituto Ortopedico Rizzoli, Via di Barbiano 1/10, 40136 Bologna, Italy; nicola.fazio@ior.it (N.F.); dvd.gravina@gmail.com (D.G.); michelina.greco@ior.it (M.G.); 4 Department of Plastic Surgery and Microsurgery, University of Cagliari, 09124 Cagliari, Italy; filippo.boriani@unica.it; 5 Unit of Anatomic Pathology, Department of Oncology, Bellaria “Carlo Alberto Pizzardi” Hospital, Via Altura 3, 40139 Bologna, Italy; 6 Scuola di Specializzazione in Ortopedia e Traumatologia, Università degli Studi di Milano, Via Festa del Perdono 7, 20122 Milano, Italy; francesca.pedrini@unimi.it; 7 IRCCS Istituto Ortopedico Galeazzi, Via Riccardo Galeazzi 4, 20161 Milano, Italy; 8 Unit of Bologna, CNR-National Research Council of Italy, Institute of Molecular Genetics “Luigi Luca Cavalli–Sforza”, Via di Barbiano 1/10, 40136 Bologna, Italy; nicoletta.zini@ior.it; 9 IRCCS Istituto Ortopedico Rizzoli, Via di Barbiano 1/10, 40136 Bologna, Italy; 10 Microbiology Section of the Department of Experimental, Diagnostic and Specialty Medicine, Azienda Ospedaliero-Universitaria di Bologna, Via Albertoni 15, 40138 Bologna, Italy; michela.paolucci3@gmail.com (M.P.); mariacarla.re@unibo.it (M.C.R.); 11 Pathology Unit, Department of Specialized, Experimental and Diagnostic Medicine, Azienda Ospedaliero-Universitaria di Bologna, Via Albertoni 15, 40138 Bologna, Italy; antonietta.derrico@unibo.it

**Keywords:** allografting, decellularization, orthopedic and maxillofacial surgery, nerve regeneration, reconstructive surgery, tissue transplantation

## Abstract

Defects of the peripheral nervous system are extremely frequent in trauma and surgeries and have high socioeconomic costs. If the direct suture of a lesion is not possible, i.e., nerve gap > 2 cm, it is necessary to use grafts. While the gold standard is the autograft, it has disadvantages related to its harvesting, with an inevitable functional deficit and further morbidity. An alternative to autografting is represented by the acellular nerve allograft (ANA), which avoids disadvantages of autograft harvesting and fresh allograft rejection. In this research, the authors intend to transfer to human nerves a novel technique, previously implemented in animal models, to decellularize nerves. The new method is based on soaking the nerve tissues in decellularizing solutions while associating ultrasounds and freeze–thaw cycles. It is performed without interrupting the sterility chain, so that the new graft may not require post-production γ-ray irradiation, which is suspected to affect the structural and functional quality of tissues. The new method is rapid, safe, and inexpensive if compared with available commercial ANAs. Histology and immunohistochemistry have been adopted to evaluate the new decellularized nerves. The study shows that the new method can be applied to human nerve samples, obtaining similar, and, sometimes better, results compared with the chosen control method, the Hudson technique.

## 1. Introduction

The annual incidence of peripheral nerve injuries (PNI) is 1:1000, with a related cost of about 150 billion dollars [1], in addition to the socio-economic costs derived from absence from work.

Characterizing up to 3.3% of traumatic injuries [2], PNI are also common in several conditions requiring replacement of nerve defects, such as oncologic and orthopedic surgeries, as well as in many other conditions requiring replacement of nerve defects in several other surgical specialties [3,4,5]. Affected patients suffer from chronic pain and/or permanent motor and sensory deficits that negatively affect their quality of life [6].

Although the peripheral nerve system displays an innate physiological regeneration property, it is not always possible to recover the nerves’ full activity. In the case of PNI, the first approach is primary neurorrhaphy, which is a direct nerve repair with epineural micro-sutures of two stumps. However, this is not feasible in the case of stump retraction or in the case of loss of tissue (gap > 2 cm) [7], where the main surgical options are autologous grafts, allogenic grafts, or nerve conduits [8].

Although the autologous graft is still considered the gold standard in PNI, its limits, such as poor availability and morbidity of the donor site, have inclined research towards other therapeutic solutions, i.e., acellular nerve allografts (ANAs). ANAs act as temporary scaffolds to regenerate axons, supporting axonal sprouting via their intact basal lamina and extracellular matrix, bypassing the need for immunosuppression by eliminating biological antigenic components.

Commercial ANAs are already available on the market. However, even if these grafts are a good surgical option, they still present some limitations (mainly related to their high cost, shipment boundary, and US donor selection criteria that differ from European criteria). Moreover, available commercial ANAs need post-manufacturing γ-ray sterilization, whose effects on tissues integrity are still controversial [9,10].

In order to avoid post-manufacturing sterilizations, preparing methods should be performed within aseptic environments, such as Class A cleanrooms. However, known decellularization techniques are not optimal for transference into clean rooms, since they involve several manipulation steps that are performed over a period longer than 4 days [11].

The authors propose a new decellularization method that allows for performing the tissue direct manipulation step within five hours, thereby making the technique sustainable in a sterile environment-based production cycle. This method has already been tested on animal models (rabbit) and the positive results obtained from these studies [12,13] have encouraged the authors to histologically validate the proposed protocol on human nerves.

This study aims to evaluate the outcomes of the new decellularization method on human nerves, in terms of histology, immunohistochemistry and microbiology, in order to prepare the field for its clinical translation.

## 2. Materials and Methods

This study was conducted following approval of local Ethical Committee (Comitato Etico Indipendente Dell’Azienda Ospedaliero-Universitaria di Bologna, Policlinico Sant’Orsola-Malpighi, Authorization 1293/2017 of 24 April 2017); a specific informed consent signed by the relative and/or legal guardian was mandatory for each donor.

### 2.1. Nerve Collection

Donors were selected based on the following criteria. Inclusion criteria: death at the authors’ institutions with the indication to undergo autoptic examination, individual age between 30 y.o. and 80 y.o. Exclusion criteria: neurodegenerative pathology, diabetic neuropathy, sepsis, HCV, HIV, metastatic neoplasia with associated paraneoplastic syndrome, meningitis. Harvesting of the nerves was conducted with the approval of the patient’s family, after explaining the method of collection and the purposes of the study.

After death, right leg sural nerves grafts were harvested within 48 h, during autoptic examination, and in aseptic conditions. For the harvesting procedure, a clean area was set up as for surgery operations in the operating room by isolating and disinfecting the area with povidone-iodine.

Samples were harvested to a maximum length of 20 cm and perineural adipose tissue was removed during the harvesting procedure. We reserved for the nerve processing and histological analysis the sural nerve segment (6–7 cm) centered at the nerve midpoint, which had a constant caliber. The sural nerve caliber at midpoint ranged between 2.4 and 2.9 mm. Small segments of the same nerves were separately collected for microbiological analyses. Finally, the harvesting site was sutured.

In total, seven different donors were available, with an average age of 67.5 years. Donor ages ranged from a minimum of 51 y.o. to a maximum of 79 y.o.

### 2.2. Experimental Scheme

Each harvested nerve tissue was split into three samples: one was treated with the innovative method, one was decellularized with the Hudson method (control method) and one was left as untreated nerve.

All nerves were then observed with Light Microscopy (LM) and Transmission Electron Microscopy (TEM) to assess removal of myelin, cells, and cell debris, and immunohistochemically tested to evaluate the degree of decellularization.

### 2.3. Decellularization Protocols

- Experimental method

Initial decellularization protocol was the same as that previously tested on rabbits [8], but was refined to be adapted to human nerves as follows:

(A) Nerves were immersed in 40 mL of phosphate-buffered saline (PBS) containing Sulfobetaine-10 (SB-10) 125 mM (SB-10, Soltec Bio Science, Beverly, MA, USA), 0.2% *v*/*v* Triton X-100 (Sigma-Aldrich S.r.l., Milan, Italy) (Triton X-200 was no longer available) and 2% *v*/*v* Pen Strep (Pen Strep, Thermo Fisher Scientific, Inc., Waltham, MA USA), incubated for 120 h at room temperature in the orbital shaker, and then frozen.
(B) On the day of manipulation, nerves were transferred to a Class A glove box to simulate sterile conditions and thawed.
(C) Nerves were rinsed three times with PBS (total 30 min) and then immersed in sterile PBS containing 0.25% Sodium Dodecyl Sulfate (SDS, Sigma- Aldrich S.r.l., Milan, Italy) for 180 min. During this phase, ultrasounds (40 Hz) were applied for 3 min every 30 min. Sonication cycles were performed with nerves soaked in 30 mL of decellularization solution sealed inside sterile 50 mL tubes, which, in turn, were immersed in a sonicator (Branson 2510 DTH Bath Sonicator; ultrasound frequency 40 Hz). During incubation, tubes were kept in agitation on a radial shaker.
(D) At the end, ANAs were rinsed three times with PBS for 30 min and immersed in 10% *v*/*v* Dimethyl Sulfoxide (DMSO) in isotonic saline solution, then frozen at −80 °C for long-term preservation.

- Hudson method (control) (adapted from [11]):

Nerves were placed in a 50 mL tube filled with PBS. All washing steps were performed at 25 °C under constant agitation. After 7 h, the PBS was removed and replaced with a solution containing 125 mM sulfobetaine-10 (SB-10) in PBS. Tubes containing the nerves in the SB-10 solution were then placed on a radial shaker for 15 h. After washing the tissues with sterile PBS for 15 min under constant agitation, nerves were soaked with a PBS solution containing 0.14% Triton X-100 (Triton X-200 was no longer available) and 0.6 mM sulfobetaine-16 (SB-16). After agitation for 24 h in a radial shaker, tissues were rinsed three times with PBS (5 min/rinse). The whole described cycle was then repeated a second time. Finally, ANAs were rinsed three times with PBS for 30 min and immersed in 10% *v*/*v* DMSO in isotonic saline solution, then frozen at −80 °C for long-term preservation.

### 2.4. Microbiology

Microbiological investigations were performed in 3 stages: T0 sampling time, T1 half process and T2 end of process.

The microbiological analyses were carried out with different bacteria broths, based on examination time: at T0 and T1, tryptic soy broth (TSB) and thioglycollate (THIOG); at T2, BacT/ALERT^®^ iFA Plus (Bio Merieux, Florence, Italy) (aerobic microorganisms) and BacT/ALERT^®^ iFN Plus (anaerobes microorganisms). iFA and iFN were adopted to simulate the release criteria required by the local competent authority to anticipate future translation to clinical fields.

### 2.5. Histology and Immunohistochemistry

Nerve and decellularized nerve fragments were fixed in 4% (vol/vol) phosphate-buffered paraformaldehyde and were paraffin-embedded for LM and immunohistochemistry; 5 µm-thick sections were mounted on slides. Tissue sections were dewaxed, rehydrated, and prepared for different stains.

Routine staining with hematoxylin–eosin (HE) (Sigma-Aldrich S.r.l., Milan, Italy) was performed to evaluate general morphology and presence of cell nuclei. Luxol Fast Blue (Sigma-Aldrich S.r.l., Milan, Italy) staining was used to analyze the presence of myelin.

Immunohistochemistry was performed in an automated stainer using 7 Ventana-purchased pre-diluted antibodies (Ventana, Tucson, AZ, USA). Antibodies were used according to standardized protocols: anti-S100 (polyclonal) highlighted Schwann cells, anti-EMA (epithelial membrane antigen, clone E29) was used for perineurium, anti-NF (neurofilament, clone 2F11) demonstrated axons and anti-Collagen IV (clone CIV22) with anti-Laminin (clone D18) stained the extracellular matrix (ECM) of the nerve.

To compare the decellularization outcomes, the following scores were adopted to assess the different nerve histological and immunohistochemical characteristics:

For endoneurium, perineurium and epineurium preservation (global nerve preservation): **badly preserved**, **moderately preserved**, **well-preserved** (for the evaluation, see Results section below).

For nuclear density: **absent** = 0; **low** < 25/10 High Powered Field (HPF); **medium** = 25–50/HPF; **high** > 50/HPF. For nuclear status: **intact** or **degenerated (nuclear debris without distinct shape)**.

For Schwann Cells density (distinct immunopositivity for S-100 both in the nucleus and cytoplasm): **absent** = 0; **low** < 25/10 High Powered Field (HPF); **medium** = 25–50/HPF; **high** > 50/HPF.

For perineurial cells density (distinct membrane immunopositivity for EMA): **absent** = 0; **low** < 2 5/10 High Powered Field (HPF); **medium** = 25–50/HPF; **high** > 50/HPF.

For axonal network: **well-preserved** (bright immunointensity, preserved shape, parallel orientation for NF) or **badly preserved** (reduced immunointensity for NF and axonal swelling).

For ECM proteins: **well-preserved** (bright, continuous and homogeneous immunointensity for Collagen IV and Laminin) or **badly preserved** (weak and dishomogeneous immunoreactivity).

### 2.6. Transmission Electron Microscopy

Nerve and decellularized nerve samples were processed for toluidine blue staining and TEM observation. Briefly, each sample was fixed with 2.5% glutaraldehyde in 0.1 M phosphate buffer, pH 7.4, post-fixed in osmium tetroxide, dehydrated in ethanol, and embedded in Epon. 0.5 µm-thick semithin cross-sections were toluidine blue-stained and used for LM analysis. Sections were observed with a Zeiss Axiophot apparatus (Carl Zeiss AG, Oberkochen, Germany); images were captured using a Nikon digital camera Digital Sight (Nikon Corporation, Tokyo, Japan). Thin cross-sections (0.1 µm) were stained with tannic acid, uranyl acetate and lead citrate, and observed with a Zeiss EM 109 transmission electron microscope. Images were captured using a Nikon digital camera Dmx 1200F and Nikon ACT-1 software.

## 3. Results

### 3.1. Microbiology

Samples were always negative at T0, T1 and T2. The only exception was the sample n.1 at T0, positive for streptococcus salivarius. However, positivity disappeared at T1, after incubation with the first solution, which contains antimicrobial agents.

### 3.2. Transmission Electronic Microscopy

**Figure 1:**
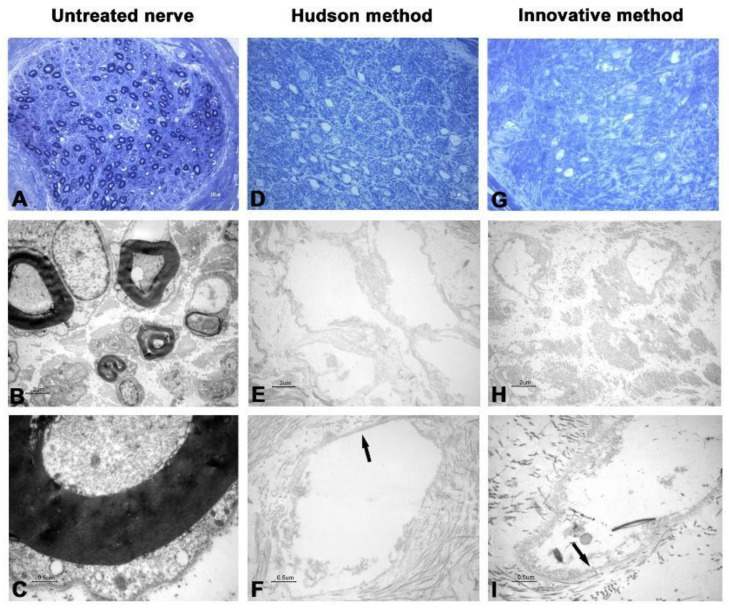
Light and electron microscopy of human nerve sample n.1. (**A**–**C**) Untreated nerve; (**D**–**F**) control decellularization method (Hudson method); (**G**–**I**) innovative decellularization method. (**A**,**D**,**G**) LM 40× of semithin cross-sections (toluidine blue, bar: 10 μm); (**B**,**E**,**H**) TEM 3000× of ultrathin cross-sections (bar: 2 μm); (**C**,**F**,**I**) TEM 12,000× at higher magnification (bar: 0.5 μm). Arrows indicate the preserved basal lamina.

**Figure 2:**
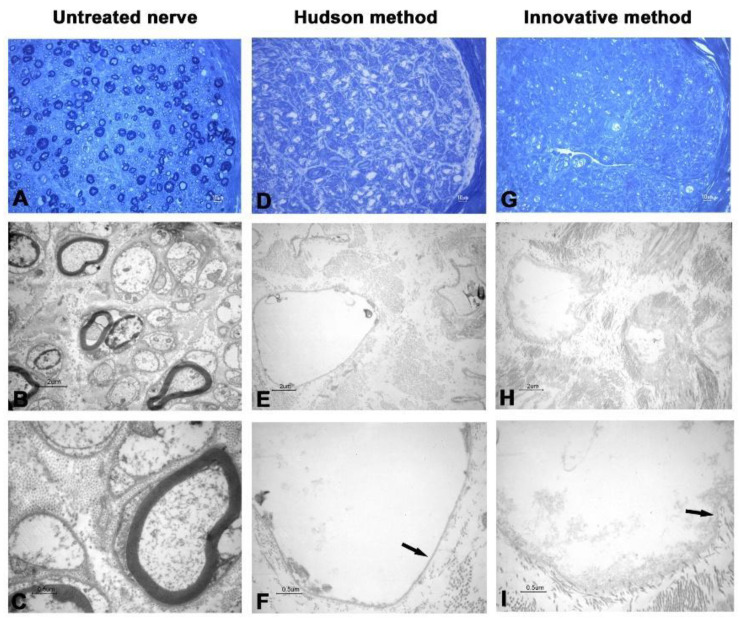
Light and electron microscopy of human nerve sample n.2. (**A**–**C**) Untreated nerve; (**D**–**F**) control decellularization method (Hudson method); (**G**–**I**) innovative decellularization method. (**A**,**D**,**G**) LM 40× of semithin cross-sections (toluidine blue, bar: 10 μm); (**B**,**E**,**H**) TEM 3000× of ultrathin cross-sections (bar: 2 μm); (**C**,**F**,**I**) TEM 12,000× at higher magnification (bar: 0.5 μm). Arrows indicate the preserved basal lamina.

In the first two nerves decellularized with the innovative method, TEM analysis showed that cellular debris was detectable inside axons, and ECM was shrunk and harshly damaged (Figure 1 and Figure 2).

**Figure 3:**
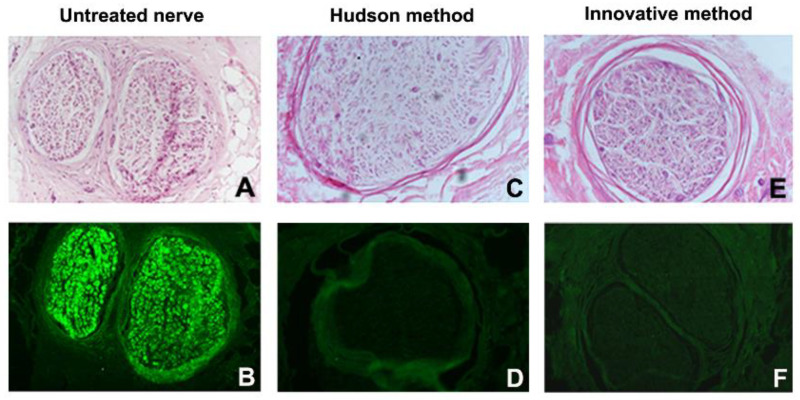
Fascicle morphology (**A**,**C**,**E**) (hematoxylin–eosin) and S-100 immunohistochemical analysis (**B**,**D**,**F**) of human nerve sample n.1. (**A**,**B**) Untreated nerve; (**C**,**D**) control decellularization method (Hudson method); (**E**,**F**) innovative decellularization method. Original magnification 20× (**A**–**F**).

**Figure 4:**
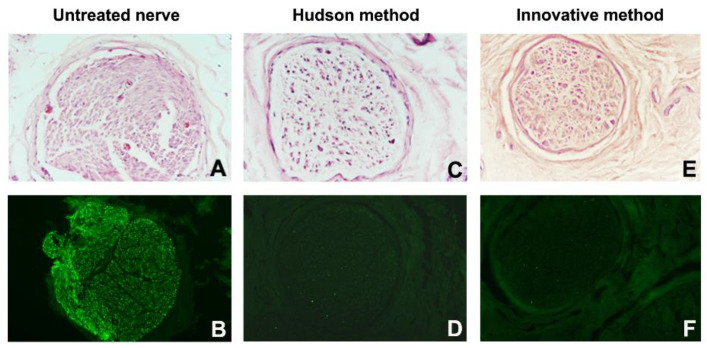
Fascicle morphology (**A**,**C**,**E**) (hematoxylin-eosin) and S-100 immunohistochemical analysis (**B**,**D**,**F**) of human nerve sample n.2. (**A**,**B**) Untreated nerve; (**C**,**D**) control decellularization method (Hudson method); (**E**,**F**) innovative decellularization method. Original magnification 20× (**A**–**F**).

Such spoiled structures were less detectable in samples prepared with the Hudson method. No evidence of residual myelin was present in either preparation. Early fluorescent immunohistochemical results showed that almost no Schwann cells residues remained in samples after treatments (Figure 3 and Figure 4), indicating that the new method is effective in terms of decellularization.

**Figure 5:**
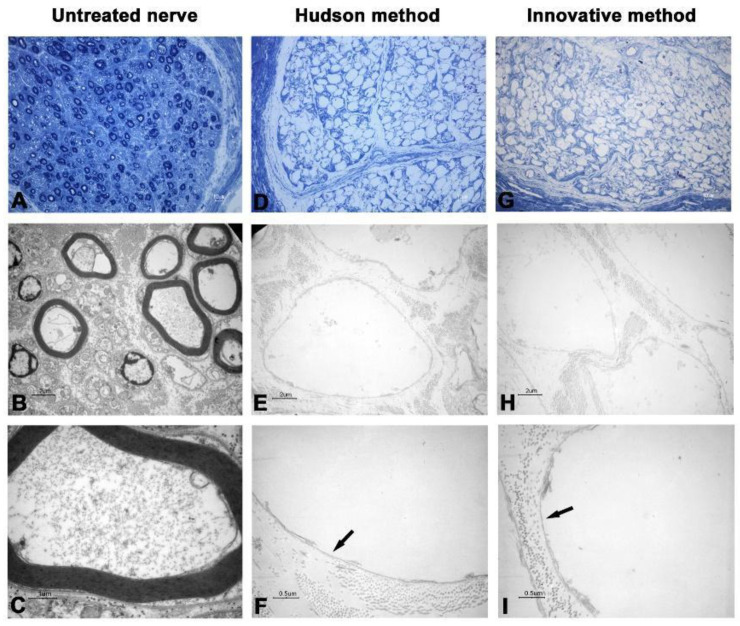
Light and electron microscopy of human nerve sample n.3. (**A**–**C**) Untreated nerve; (**D**–**F**) control decellularization method (Hudson method); (**G**–**I**) innovative decellularization method. (**A**,**D**,**G**) LM 40× of semithin cross-sections (toluidine blue, bar: 10 μm); (**B**,**E**,**H**) TEM 3000× of ultrathin cross-sections (bar: 2 μm); (**C**,**F**,**I**) TEM 12,000× at higher magnification (bar: 0.5 μm. Arrows indicate the preserved basal lamina.

**Figure 6:**
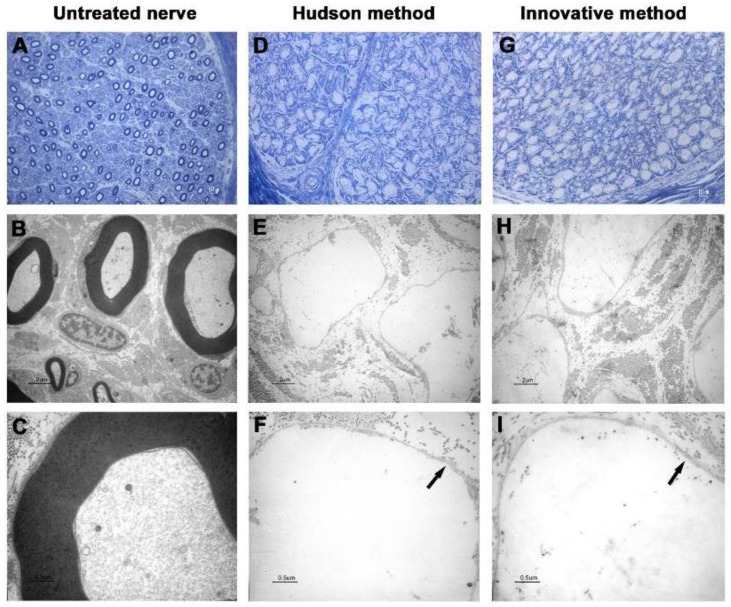
Light and electron microscopy of human nerve sample n.6. (**A**–**C**) Untreated nerve; (**D**–**F**) control decellularization method (Hudson method); (**G**–**I**) innovative decellularization method. (**A**,**D**,**G**) LM 40× of semithin cross-sections (toluidine blue, bar: 10 μm); (**B**,**E**,**H**) TEM 3000× of ultrathin cross-sections (bar: 2 μm); (**C**,**F**,**I**) TEM 12,000× at higher magnification (bar: 0.5 μm). Arrows indicate the preserved basal lamina.

**Figure 7:**
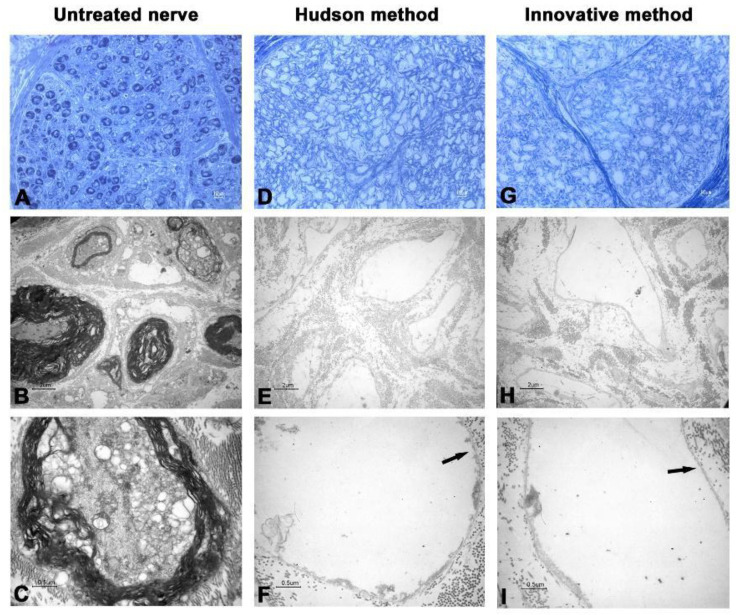
Light and electron microscopy of human nerve sample n.7. (**A**–**C**) Untreated nerve; (**D**–**F**) control decellularization method (Hudson method); (**G**–**I**) innovative decellularization method. (**A**,**D**,**G**) LM 40× of semithin cross-sections (toluidine blue, bar: 10 μm); (**B**,**E**,**H**) TEM 3000× of ultrathin cross-sections (bar: 2 μm); (**C**,**F**,**I**) TEM 12,000× at higher magnification (bar: 0.5 μm). Arrows indicate the preserved basal lamina.

Following the hypothesis that ultrasound cycles were too aggressive and spoiling the integrity of treated nerves, two modifications were applied to initial protocol. More precisely, initial incubation period was increased from 48 h to 120 h and sonication time was changed from 5 min to 3 min per cycle. This modification was first applied to sample n.3 (Figure 5), improving both clearance of cellular fragments and histomorphological integrity of ECM. At TEM, no major deteriorations of ECM were observable, axons showed no evidence of residual cells and myelin, and basal lamina was preserved. Images obtained from other samples were comparable (Figure 6 and Figure 7).

### 3.3. Histology and Immunohistochemistry

**Figure 8:**
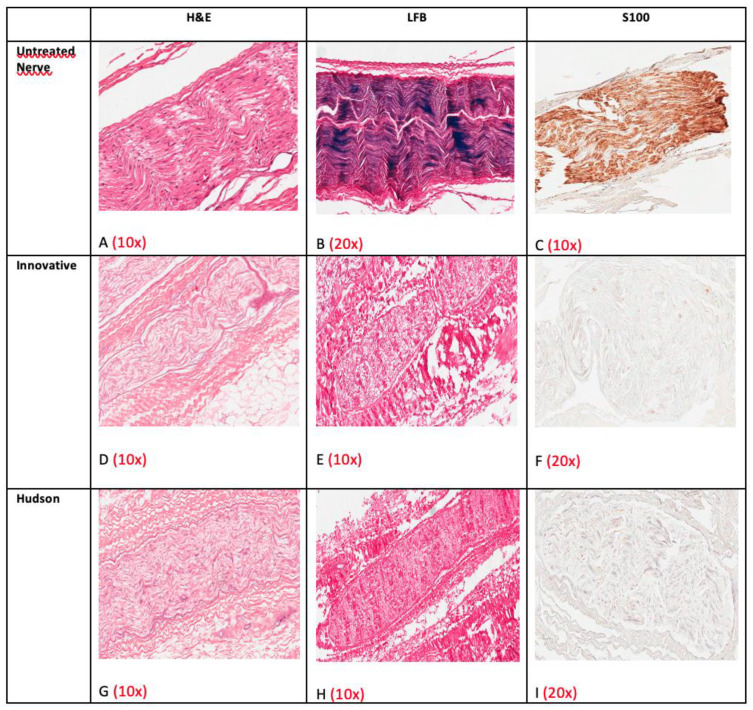
Example of structural and cellular comparison between the nerves before (**A**–**C**) and after decellularization: innovative method (**D**–**F**) and Hudson method (**G**–**I**). Note the absence of well-preserved nuclei in (**D**) and the presence of nuclear debris in (**G**).

General morphology was evaluated with HE staining. Native nerves consisted of three distinct and well-preserved compartments: endoneurium composed of axons and their accompanying Schwann cells; perineurium consisting of concentrically disposed flattened and polygonal cells separated by a thin layer of collagen; epineurium composed of fibrous and adipose tissue with small arteries, veins and lymphatics (Figure 8A–C).

After decellularization, the epineurium and the perineurium fibrous tissue were well-preserved in both groups, but no more intact cell nuclei were detectable (Figure 8D–I). Structures of endoneurium were generally preserved, but a small number of degenerated nuclei and chromatin debris were still noticeable in endoneurium (Figure 8G).

Luxol Fast Blue revealed removal of myelin in both decellularization groups (Figure 8E,H).

S100 and EMA immunohistochemical staining confirmed these observations: in native nerves, Schwann and perineurial cells were easily highlighted (Figure 8C), whereas after decellularization no distinct positivity could be noticed (Figure 8F,I).

**Figure 9:**
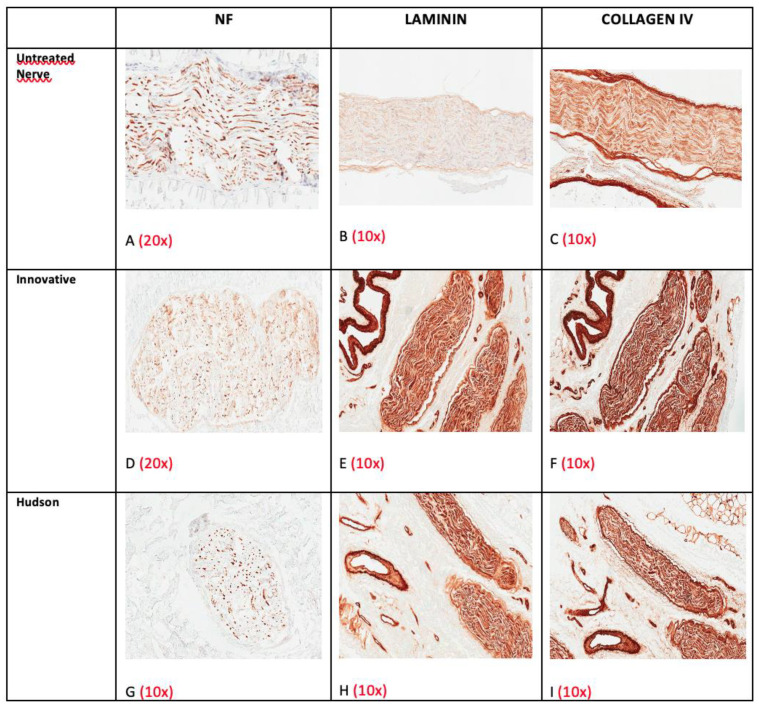
Example of axonal and ECM preservation in nerves before (**A**–**C**) and after decellularization: innovative method (**D**–**F**) and Hudson method (**G**–**I**). Note the increasing density and the different distribution of the ECM after decellularization.

Native nerves contained moderately preserved axons (Figure 9A) and preserved ECM (Figure 9B,C), whereas decellularized groups retained only traces of axonal proteins and very well-preserved ECM components, as confirmed by the immunohistochemical staining for Laminin and Collagen type IV (Figure 9D–I). The latter two stainings were often more evident in decellularized samples; presumably, after decellularization, proteins were more unmasked for antibody binding.

In five specimens (cases 1, 2, 3, 6, 7) the innovative method revealed a higher degree of decellularization than the Hudson process with a well-preserved structure of the nerve (Figure 8D,G). In two specimens (cases 4, 5) the Hudson process had better results (Table 1).

## 4. Discussion

The use of ANAs for peripheral nerve reconstruction has recently spread, as shown by recent medical literature. Isaacs and coworkers [14] have implanted ANAs in combination with nerve connectors in rats and Hong and coworkers [15] have successfully tested ANAs in an animal setting for such a challenging problem as painful neuroma. ANAs were also efficaciously employed in a clinical context for human trigeminal nerve [16] and upper extremities reconstructions [17] and they have been successfully utilized for brachial plexus reconstruction by Li and collaborators [18].

Enhancing ANAs in animal models through either microsurgical techniques supercharging [19] or adipose stem cells-based seeding [20] promotes neuroregeneration.

In the present study, authors histologically validated an effective decellularization method able to preserve ECM, whose role in conditioning nerve regeneration is essential. The method is also microbiologically safe and therefore easy to translate to aseptic environments. The method, previously optimized in vitro and in vivo as presented by this research group [12], can be applied on human nerve samples, obtaining similar and sometimes even better results compared with the Hudson technique, despite an insufficient number of samples for statistically significant difference.

The first step was to use different ancillary tests to confirm the success of the decellularization method.

LM observation of sections stained with HE revealed degeneration of cells, absence of myelin, and maintenance of both morphological characteristics and basal lamina, with interstitial endoneurium preservation.

Immunohistochemistry and TEM confirmed that ECM components were preserved and highlighted that cellular debris, detectable at LM, corresponded neither to intact nuclei nor to cytoplasmic structure.

The second step was to compare results obtained with the innovative protocol against those obtained with the Hudson technique.

As shown in Table 1, the innovative method revealed a better degree of decellularization than the Hudson process in 5 out of 7 cases (cases 1, 2, 3, 6, 7), whereas in the remaining 2 out of 7 cases (cases 4, 5) the Hudson process had slightly better results. The innovative technique revealed histologically complete nerve decellularization in case 3 (see also Figure 8D). As stated before, this result is encouraging, but not statistically significant.

Routine staining with HE revealed that in cases 1, 2, 6, and 7, density and distribution of the debris were higher in Hudson-treated nerves than in innovatively treated specimens. In addition, TEM did not detect any intact nuclear or cytoplasmic components.

TEM observation did not confirm any intact axonal structures after both decellularization treatments in all specimens.

As also shown by other groups [21], in this experimental protocol, ECM components were well-preserved after both decellularization techniques; the innovative method revealed a better degree of ECM preservation in 2 out of 7 cases (cases 4, 7 = 30%).

Reducing the protocol duration to only one day of manipulation within the sterile environment and limiting the tissue handling time to stay under the 5 h of a common aseptic working session renders this technique as compatible as possible with the requirements of cleanroom manufacturing. In addition, as Azhim et al. [22] suggested, limiting tissue exposure can shrink costs for the whole process and help avoid post-manufacturing sterilization, in particular, γ-irradiation, a procedure considered controversial and supposedly damaging to ANA stromal integrity [9,10]).

The new proposed protocol employs low concentrations of multiple chemical detergents, allowing a more extensive detergent clearance after decellularization [23,24,25], thus providing the graft with a more suitable environment for cellular proliferation and colonization. As shown in Figure 1 and Figure 2, the 5 min sonication cycle appears to have detrimental effects on nerve histomorphological integrity. After the mentioned variation of exposure to sonication, from 5 to 3 min per cycle, nerve ECM ultrastructure was nearly clear from damage, a common drawback reported in other previously published studies [26,27].

Sonication appears to be a correct coupling for and completion to freeze-thawing. According to the findings of Szynkaruk et al. [28], freeze-thawing clears cells only if a long procedure is employed, such as in the Hudson method; this work also demonstrated that coupling sonication to freeze-thawing achieved a cell-free allograft within a reasonable time. Moreover, based on negative microbiology results obtained at various stages of nerve processing, the final product could be considered sterile, thus ready for its clinical employment in nerve reconstructions. For translation to clinical practice, production of the decellularized nerve grafts proposed by the authors will be feasible in a cleanroom Class A or ISO 1 as defined by ISO 14644 classification.

## 5. Conclusions

Our protocol of decellularization, previously studied in vivo to functionally compare it with the Hudson method and now optimized in vitro on human nerves, has the potential to achieve an aseptic and entirely decellularized nerve tissue graft, within a shorter processing time compared to pre-existing protocols, and without the need of post-production γ-ray sterilization. Moreover, the whole method has the great advantage of being easily performed inside certified aseptic environments, such as clean rooms. Europe follows the principle that “*the human body and its parts shall not give rise, as such, to financial gain*” and only Authorized Tissue Banks can distribute donated tissues; however, to date, none of these structures produce and distribute cadaver donor Acellular Nerve Allografts for surgical reconstructions. This work represents the first step to provide a novel, safe and inexpensive tool to be used by European Tissue Banks to democratize the employment of nerve tissue transplantation for nerve injuries reconstruction.

A limitation of the study is the absence of a clinical translation, even in the form of a pilot study, to assess the implantation safety of ANAs. Therefore, after performing two reviews of the literature [29,30] and the two above-mentioned preclinical studies [12,13], our research group is now planning a preliminary clinical validation of human nerve grafts obtained with the method described.

## Figures and Tables

**Table 1:** Different decellularization techniques based on morphological and immunohistochemical results (abbreviations: EMA, epithelial membrane antigen; NF, neurofilament; ECM, extracellular matrix). Scores for evaluation are described in the section “Materials and methods: Histology and immunohistochemistry”.

Cases	Decellularization Techniques	Parameters for the Evaluation of Decellularization Process					Evaluation of Neural Support Structure	Results
		Global Nerve Preservation	Nuclear Density and Status	Schwann Cells (S100)	Perineural Cells (EMA)	Axons Preservation (NF)	ECM Preservation (Laminin and Collagen IV)	
1	Native Innovative Hudson	well well well	High, intact Low, degenerated Medium, degenerated	High Absent Absent	High Absent Absent	Well Badly Badly	Well Well Well	Innovative better than Hudson
2	Native Innovative Hudson	well well well	High, intact Low, degenerated Medium, degenerated	High Absent Absent	High Absent Absent	Badly Badly Badly	Well Well Well	Innovative better than Hudson
3	Native Innovative Hudson	well moderately moderately	High, intact Absent Low, degenerated	High Absent Absent	High Absent Absent	Well Badly Badly	Well Well Well	Innovative better than Hudson (Innovative with complete decellularization)
4	Native Innovative Hudson	well well moderately	High, intact Medium, degenerated Low, degenerated	High Absent Absent	High Absent Absent	Badly Badly Badly	Well Well Bad	Hudson better than Innovative but without ECM preservation
5	Native Innovative Hudson	well well well	High, intact Medium, degenerated Low, degenerated	High Absent Absent	High Absent Absent	Badly Badly Badly	Well Well Well	Hudson better than Innovative
6	Native Innovative Hudson	well well well	High, intact Low, degenerated Medium, degenerated	Medium Absent Absent	Medium Absent Absent	Well Badly Bad	Well Well Well	Innovative better than Hudson
7	Native Innovative Hudson	moderately moderately moderately	High, intact Low, degenerated Medium, degenerated	High Absent Absent	High Absent Absent	Well Badly Badly	Well Well Badly	Innovative better than Hudson

## Data Availability

Data available on request from the Authors.
